# P-1780. Association between Neurocysticercosis and Mental Health Outcomes in School-Aged Children in Rural Sichuan, People’s Republic of China

**DOI:** 10.1093/ofid/ofaf695.1949

**Published:** 2026-01-11

**Authors:** Overbeck Christian Takou Mbah, Yuju Wu, John Openshaw

**Affiliations:** Stanford University, Stanford, California; Sichuan University, Chengdu, Sichuan, China; Stanford University, Stanford, California

## Abstract

**Background:**

Neurocysticercosis (NCC) is a tissue-invasive parasitic disease of the central nervous system caused by *Taenia solium*, a pork tapeworm endemic to low-resource regions such as rural China. NCC can cause headaches, cognitive deficits, and seizures, but its impact on mental health remains poorly characterized.

**Methods:**

We investigated the association between NCC and child mental health in rural Sichuan, where our team has previously documented high T. solium prevalence among primary school children. We hypothesized that children with NCC would report more mental health symptoms than those without. We enrolled 3,029 school-aged children from three counties. We conducted cross-sectional surveys to assess *T. solium* exposure, medical history, and mental health outcomes. We collected cysticercosis antibody serum titers from children. Due to limited MRI access, we established a modified case definition of probable NCC using guideline-based criteria that excluded imaging. Chi-square and Fisher’s exact tests compared children with vs. without NCC. Multivariable logistic regression will assess associations between NCC and mental health symptoms.

**Results:**

Probable NCC prevalence was 4.2%. NCC was significantly associated with Tibetan ethnicity (p = 0.01), boarding school status (p = 0.03), pig ownership (p = 0.008), pigs in defecation areas (p = 0.002), and lack of handwashing before eating (p = 0.04). NCC was also strongly associated with cysticercosis seropositivity and MRI-confirmed NCC (p < 0.001 for both). Among the full sample, 2,599 children (85.8%) reported anxiety symptoms, 1,981 (65.4%) reported social isolation symptoms, 1,516 (50%) reported difficulty coping in tough situations, 1,013 (33.4%) reported poor concentration, 949 (31.3%) reported emotional lability, and 677 (22.4%) reported suicidal ideation.

**Conclusion:**

NCC and mental health symptoms are common among children in rural Sichuan. Several demographic, environmental, and behavioral factors were significantly associated with NCC. These preliminary findings suggest the need for integrated parasitic disease control and mental health screening strategies in endemic, resource-limited settings.

**Disclosures:**

All Authors: No reported disclosures

